# Generative design of crystal structures by point cloud representations and diffusion model

**DOI:** 10.1016/j.isci.2024.111659

**Published:** 2024-12-20

**Authors:** Zhelin Li, Rami Mrad, Runxian Jiao, Guan Huang, Jun Shan, Shibing Chu, Yuanping Chen

**Affiliations:** 1 School of Physics and Electronic Engineering, Jiangsu University, Zhenjiang, Jiangsu 212013, P.R. China; 2 Jiangsu Engineering Research Center on Quantum Perception and Intelligent Detection of Agricultural Information, Zhenjiang 212013, China

**Keywords:** Natural sciences, Physics, Computer science, Materials science

## Abstract

Efficiently generating energetically stable crystal structures has long been a challenge in material design, primarily due to the immense arrangement of atoms in a crystal lattice. To facilitate the discovery of stable materials, we present a framework for the generation of synthesizable materials leveraging a point cloud representation to encode intricate structural information. At the heart of this framework lies the introduction of a diffusion model as its foundational pillar. To gauge the efficacy of our approach, we employed it to reconstruct input structures from our training datasets, rigorously validating its high reconstruction performance. Furthermore, we demonstrate the profound potential of point cloud-based crystal diffusion (PCCD) by generating materials, emphasizing their synthesizability. Our research stands as a noteworthy contribution to the advancement of materials design and synthesis through the cutting-edge avenue of generative design instead of conventional substitution or experience-based discovery.

## Highlights

• We present a materials generation framework for bulk materials based on diffusion model
• We use point cloud presentation to encode atomic position information
• We generate batches of crystals and many of them have the potential for stability
• We validate our results with first-principles calculations

## Graphical abstract

**Figure:**
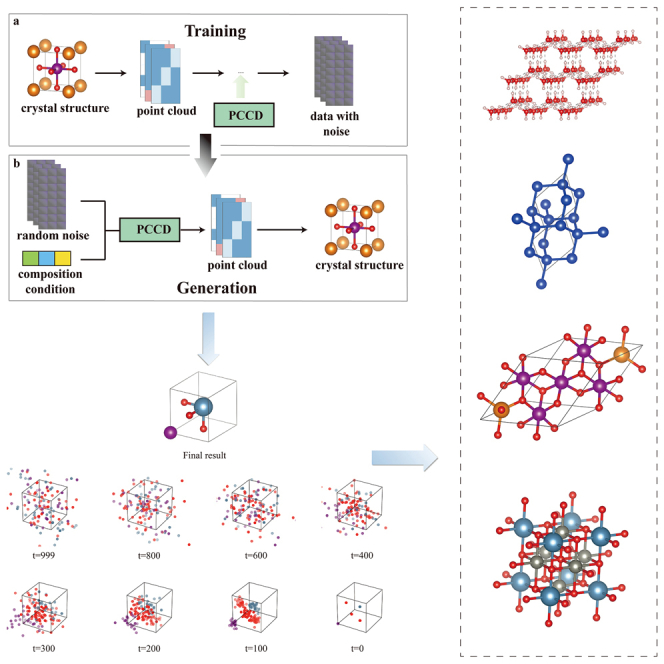

## Introduction

The continuous advancement of technology hinges significantly on the development of material science, making it essential to unravel the complex relationships between molecular or crystal structures and their properties. Currently, two main methods are used for designing crystal structures: altering existing materials using scientific intuition and empirical principles or global optimization algorithms^1^ and mining material databases with the Materials Project (MP),^2^ known as high-throughput virtual screening,^3^ which has shown great success in various applications. However, the computational expense associated with density functional theory (DFT) calculations renders an exhaustive search of the theoretical material space infeasible.^4^ In recent years, there has been a notable surge in research dedicated to harnessing artificial intelligence (AI) for the exploration of undiscovered materials.^5^^,^^6^^,^^7^^,^^8^^,^^9^^,^^10^ However, within the field of crystallography, the predominant application of machine learning (ML) techniques is focused on predicting material properties, such as composition, band gap, or formation energy.^11^^,^^12^^,^^13^ Consequently, the utilization of ML algorithms for crystal generation remains relatively nascent, underscoring the pressing need for the further development of artificial intelligence–generated content (AIGC) within the realm of crystallography.

In the field of material exploration, generative models have been proven to be particularly effective.^7^ Over the past few years, two fundamental models have been widely applied: the generative adversarial network (GAN)^14^ and the variational autoencoder (VAE).^15^ Currently, an array of studies has been dedicated to structure generation, drawing on the capabilities of these two models. An example is the study conducted by Jordan Hoffmann et al.*,*^16^ in which voxel representation was employed for crystals, a VAE was utilized for voxel data generation, and a U-Net model was subsequently applied for voxel classification. Zekun Ren et al.^5^ employed VAE for the reverse design of materials. Kim et al.^7^ utilized a GAN model to explore structures within the Mg-Mn-O ternary system, while Baekjun Kim et al.^8^ employed a Wasserstein generative adversarial network (WGAN) in their quest to discover crystalline porous materials. These research endeavors highlight the versatility and promise of generative models in the context of material discovery and design.

Recently, there has been a significant emergence of models for generating generic crystal structures. A notable example is the Crystal Diffusion Variational Autoencoder (CDVAE) developed by Tian Xie et al.,^6^ which successfully integrates a diffusion model with a VAE for crystal generation. Furthermore, the Cond-CDVAE model^17^ extends this approach by allowing the incorporation of user-defined material and physical parameters, such as composition and pressure. Another major breakthrough in this domain is MatterGen,^18^ which is capable of generating stable and materials with specified chemical compositions, symmetries, and mechanical, electronic, and magnetic properties.

Nevertheless, most models address the challenge of how to improve the quality of generation results.^19^ Jonathan Ho et al.^20^ introduced a generative model known as the denoising diffusion probabilistic model (DDPM). Notably, various research teams, such as OpenAI,^21^^,^^22^ NVIDIA^23^ and Google,^24^ have achieved significant breakthroughs in the application^25^ of this model. Considering its excellent generative capability, we aim to investigate the latent potential of this model in the domain of structure generation and its potential to enhance the creative aspects of the model. Additionally, to minimize computational expenses and tailor diffusion modeling, we propose a point cloud representation^26^ to encode atom sites, element information, and lattice constants.

In this paper, we introduce a streamlined deep learning framework for crystal generation: point cloud-based crystal diffusion (PCCD). To test the model’s reliability, we intentionally added noise to our dataset and then used the PCCD to reconstruct the majority of the inputs with only minor deviations. Furthermore, we calculated the energy above hull (E_hull_) per atom for a set of crystal structures generated by PCCD, revealing that many of these structures had low energy values, indicating their potential significance. Furthermore, our analysis revealed structures not in the database or with a stable phonon structure, emphasizing the ability of PCCD to generate potentially significant crystal structures.

## Results and discussion

### Reconstruction

**Figure 1:**
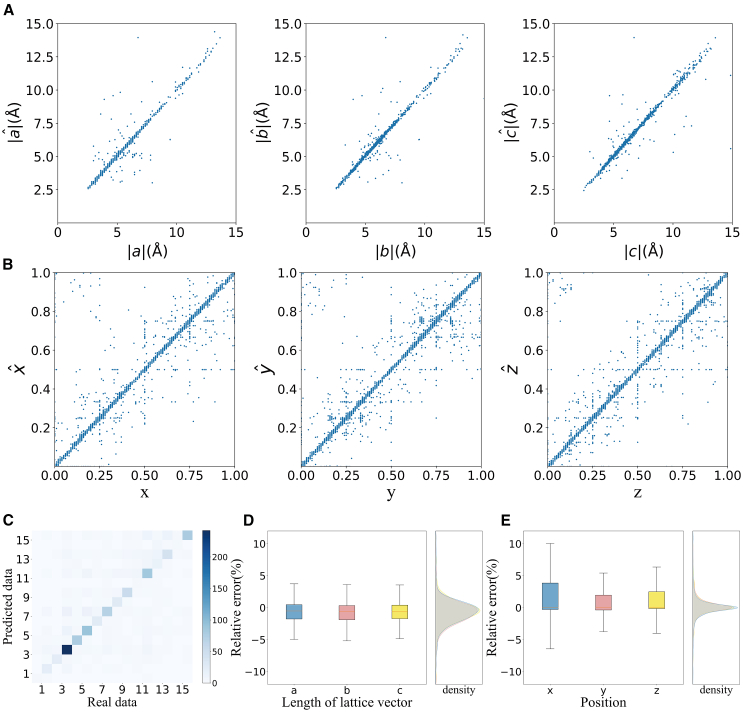
Reconstruction results of the PCCD (A) The parity plots for lattice lengths of reconstructed materials and original materials. (B) The parity plots for the atomic positions of the reconstructed materials and original materials. (C) Heatmap of the atom number relationship between the reconstructed materials and original materials. (D) Boxplot of the lattice length relative error with density distribution. (E) Boxplot of the relative errors of the atomic x, y, and z coordinates with respect to the density distribution.

In the PCCD, the training of the diffusion model involves the incremental addition of noise, with the model essentially learning how to peel noise from the corrupted data. In an ideal scenario, saving the data from the database, along with the added noise, should enable the eventual reconstruction of these original data without noise. To validate the model’s effectiveness, we selected a batch of structures from the database as the test set and performed 1000 iterations of noise to obtain and store the results. These noise-augmented results were then used as inputs for the PCCD instead of true random numbers. In theory, 1000 times of noise should be removed, and the data should be restored. To ensure accurate atomic site matching, the statistics presented are based on 868 samples, as only structures with matching atom counts can be compared. For the purpose of predicting atomic coordinates, we align each atom in the predicted crystal structure with its counterpart in the original crystal, given that both structures have the same total number of atoms. The distance between each atom in one structure and each atom in the other structure is calculated, taking into account translational symmetry. This symmetry allows atoms in the original crystal to be matched with atoms in adjacent cells of the predicted crystal, effectively aligning coordinates such as (0,0,0) in one structure with (1,1,1) in the other when the distance is zero. Finally, the Greedy Algorithm is employed to perform the matching after all distances have been determined. We then compared the restored data to the original dataset, as illustrated in Figure 1 (with detailed information provided in Table 1), providing a robust assessment of the model’s reconstruction performance. This experiment serves as a rigorous validation of the model’s capabilities.

**Table 1:** Data details for reconstruction results

	a	b	c	x	y	z
Efficiency	88.48%	90.55%	89.40%	70.83%	71.60%	72.02%
Upper limits	3.81%	3.66%	3.57%	10.05%	5.44%	6.44%
Lower limits	−5.12%	−5.23%	−4.90%	−6.50%	−3.84%	−4.09%

Efficiency calculations according to the boxplots in Figures 1D and 1E. The upper and lower limits are shown for the boxplots in Figures 1D and 1E, respectively.

Given that we did not specify the number of atoms in the PCCD, the accuracy of predicting the atom count serves as a direct indicator of the model’s performance. In Figure 1C, a heatmap illustrates the relationship between the sum of atoms in the original data and the data predicted by the PCCD. Notably, a clear diagonal line represents accurate predictions, and the accuracy rate is 67.81% (868 out of 1280 samples).

Among these 868 samples with accurately predicted atom counts, we further calculated the relative errors for the lattice length of each corresponding atom, where |a|,|b|,|c| are the original lattice lengths and $\left|\hat{a}\right|,\left|\hat{b}\right|,\left|\hat{c}\right|$ are the predicted lattice lengths (as shown in Figure 1A) and their coordinates (depicted in Figure 1B). A significant portion of these errors is visibly clustered around the y=x line. To gain deeper insights into their distribution, we conducted a detailed analysis using boxplots for both aspects, as presented in Figures 1D and 1E. Figure 1D displays the boxplot for the relative errors of the lattice parameters a, b, and c. The acceptable ranges for a, b, and c typically fall within upper limits of 3.81%, 3.66%, and 3.57%, respectively, and lower limits of −5.12%, −5.23%, and −4.90%, respectively. The effective rates for these parameters are as follows: 88.48% for lattice parameters a, 90.55% for lattice parameters b, and 89.40% for lattice parameters c. Alongside the boxplots, the kernel density function plots help illustrate the concentration of the data. In Figure 1E, we present the boxplot for the relative errors of the x, y, and z coordinates for each atom. The typical acceptable ranges for x, y, and z coordinates fall within the upper limits of 10.05%, 5.44%, and 6.44%, respectively, and lower limits of −6.50%, −3.84%, and −4.09%, respectively. The effective rates for these coordinates are calculated as 70.83% for x, 71.60% for y, and 72.02% for z. Most of the errors are relatively small, and they can be readily corrected during DFT geometry optimization. This analysis indicates that the framework is already functional and effective. However, we also conducted a more in-depth analysis to explore the objective factors that may influence model errors.

A significant contributing factor to the performance limitations of PCCD lies in the preprocessing stage before training. To facilitate the normalization of lattice vectors and enhance reversibility, all lattice vector data (data in the 3rd channel) were divided by 15. Consequently, the model’s capacity was restricted to generating values within the range of −1 to 1, which, in turn, led to a limitation in predicting lattice vectors. Specifically, the model could predict only lattice vectors with a maximum length of 15Å. As a consequence, structures featuring lattice vectors exceeding the maximum could not be accurately predicted. Upon calculating the relative errors for the lattice lengths of each atom across all samples (a total of 1280), we observed less favorable outcomes due to this limitation. The boxplots vividly illustrate that the effective rates for lattice vector length were only 73.98% for the ‘a’ lattice parameter, 75.39% for ‘b’, and 71.64% for ‘c’. This highlights the significant correspondence between the errors in both the atom counts and lattice vector length predictions.

Notably, many of these errors were associated with structures featuring at least one lattice vector longer than the maximum. This suggests a substantial interrelation between the atom count and lattice vector predictions, despite their presence in different data channels. Furthermore, the comparison of the x,y and z positions of atoms, as shown in the three figures in Figure 1B, reveals that a portion of the data clustered around the position (0,1). These data points were excluded when calculating statistics, as they were considered erroneous. However, it is important to note that crystal cells are periodic, and such data points are essentially equivalent to (0,0) or (1,1). This periodicity factor contributes to lower accuracy in the statistical analysis.

**Figure 2:**
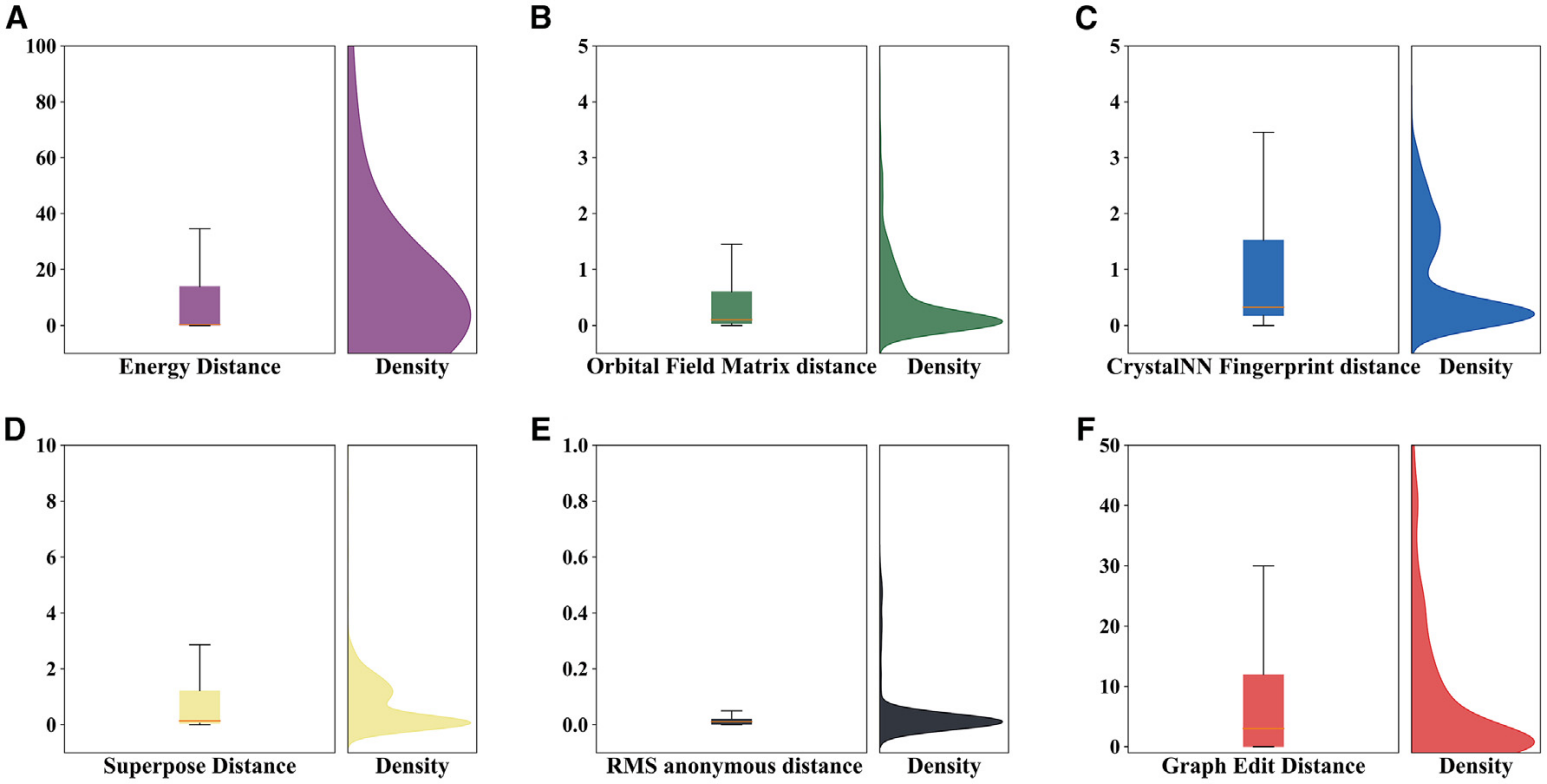
Statistics for matched structures (A–F) Boxplots and kernel density functions of the Energy distance (A), Orbital Field Matrix distance (B), CrystalNN Fingerprint distance (C), Superpose distance (D), RMS Anonymous distance (E) and Graph Edit distance (F) for matched crystals.

In order to describe the matching relationships between structures more accurately, we selected several statistical metrics that are suitable for crystal structure prediction (CSP).^27^ For each pair of crystals composed of a reconstructed structure and its original structure, we calculated their energy distance (Figure 2A), orbital field matrix distance (Figure 2B), CrystalNN fingerprint distance (Figure 2C), superpose distance (Figure 2D), RMS anonymous distance (Figure 2E) and graph edit distance (Figure 2F). Boxplots and kernel density function graphs can reflect their distribution situation. Figure 2 presents the outputs of the statistics, from which we can deduce that, for the energy distance, the reconstruction process of the model consists of obtaining values close to 0, proving the efficiency of the crystal reconstruction process. The boxplot in Figure 2A shows that the largest portion of the reconstructed data are near to 0. Figure 2B illustrates the orbital field matrix distance, where the density of the reconstructed data shows a peak at approximately 0; here, we can conclude that the reconstruction process is efficient. The CrystalNN fingerprint distance is an ML-based approach that investigates the number of neighboring atoms in the same or similar crystal structures; hence, Figure 2C shows the repartition of the data, which proves the similarity of the reconstructed data. Figure 2D shows the superpose distance, which reflects the efficacity of the model in the training process, where these metrics are used to compare the similarity of the periodic structure. Illustrating the RMS anonymous distance in Figure 2E is an absolute shoring to our model where we constate the similarity of the reconstructed data in these evaluation metrics and prove the model capacity. By exploring Figure 2F and the last performance metrics that we chose for assessing our work, the graph edit distance, we can also deduce the success of the reconstruction process due to the output of this metric, where we compare the number of edges and nodes; hence, we notice the peak at 0 to prove the structural similarity in terms of reconstruction.

### Generated results

**Figure 3:**
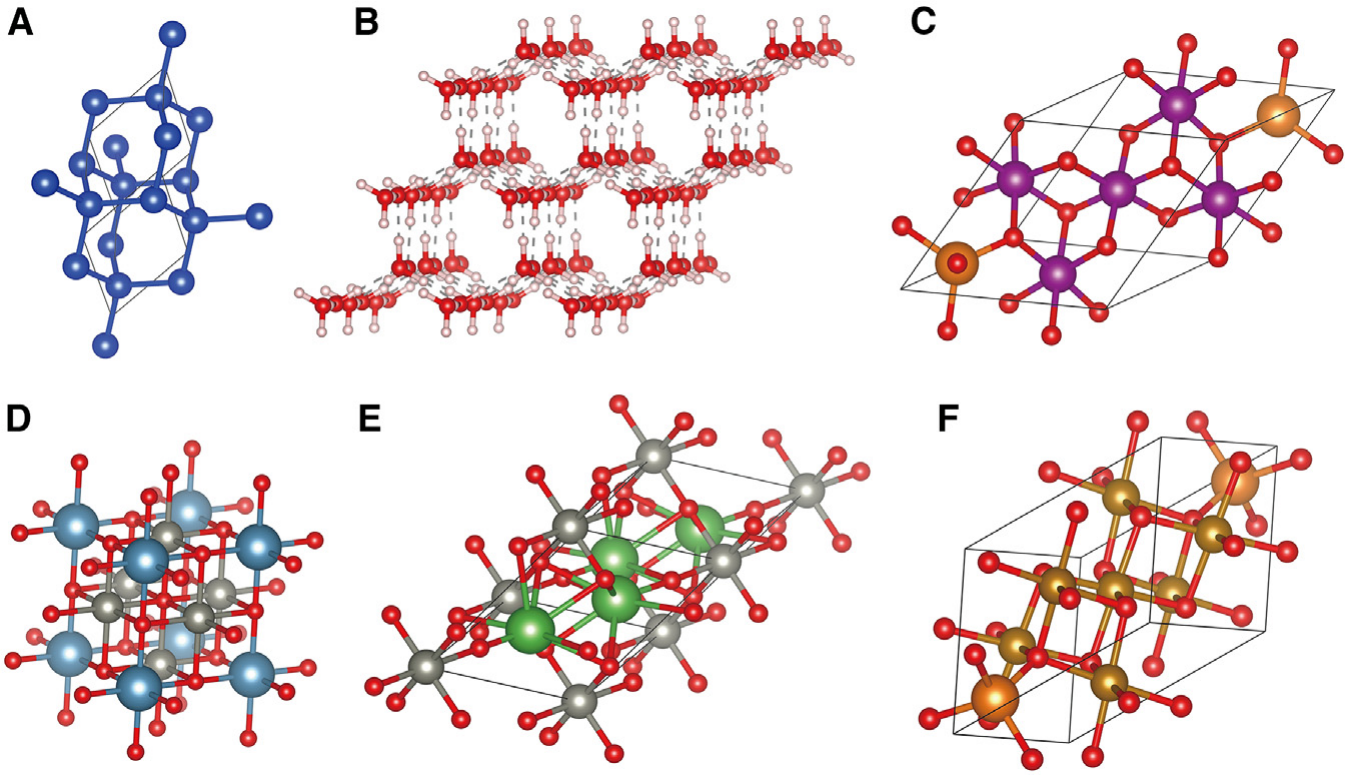
Examples of generated crystals (A) Sample of the predicted data Si system. (B) A sample of the predicted data 3×3 supercell for the H-O system (H_2_O). (C) Predicted data of the unit cell for the Mg-Mn-O system (Mg_2_Mn_3_O_8_). (D) Unit cell generated for CaZn_3_O_4_. (E) Unit cell generated for La_2_ZnO_4_. (F) Unit cell generated for MgFe_2_O_4_.

In comparison to models tailored for specific material components, such as the Mg-Mn-O or V_x_O_y_ systems,^7^^,^^28^ PCCD demonstrates superior generalization capabilities. This means that we can effectively generate crystal structures composed of any combination of elements, provided that the total number of elements does not exceed three. As depicted in Figure 3, PCCD enables the generation of unary systems (Figure 3A), binary systems (Figure 3B), and ternary systems (Figure 3C), demonstrating its versatility and broad applicability. These findings also prove the diversity of this framework (Figures 3D–3F).

For statistical validation and comparative analysis with other models, we generated three distinct batches of structures. The first batch (batch #1) consists of all possible elements excluding noble gases and radioactive elements. The second batch (batch #2) comprises rare earth elements, alkaline earth elements, transition metals, and oxygen, selected based on their ability to display characteristic properties that set them apart. The third batch (batch #3) comprises only commonly used elements. Following an initial screening process, we identified 1809, 746, and 120 structures in each batch, respectively. Of these, 1680 (92.87%), 669 (89.68%), and 108 (90%) structures were not exist in database.

**Figure 4:**
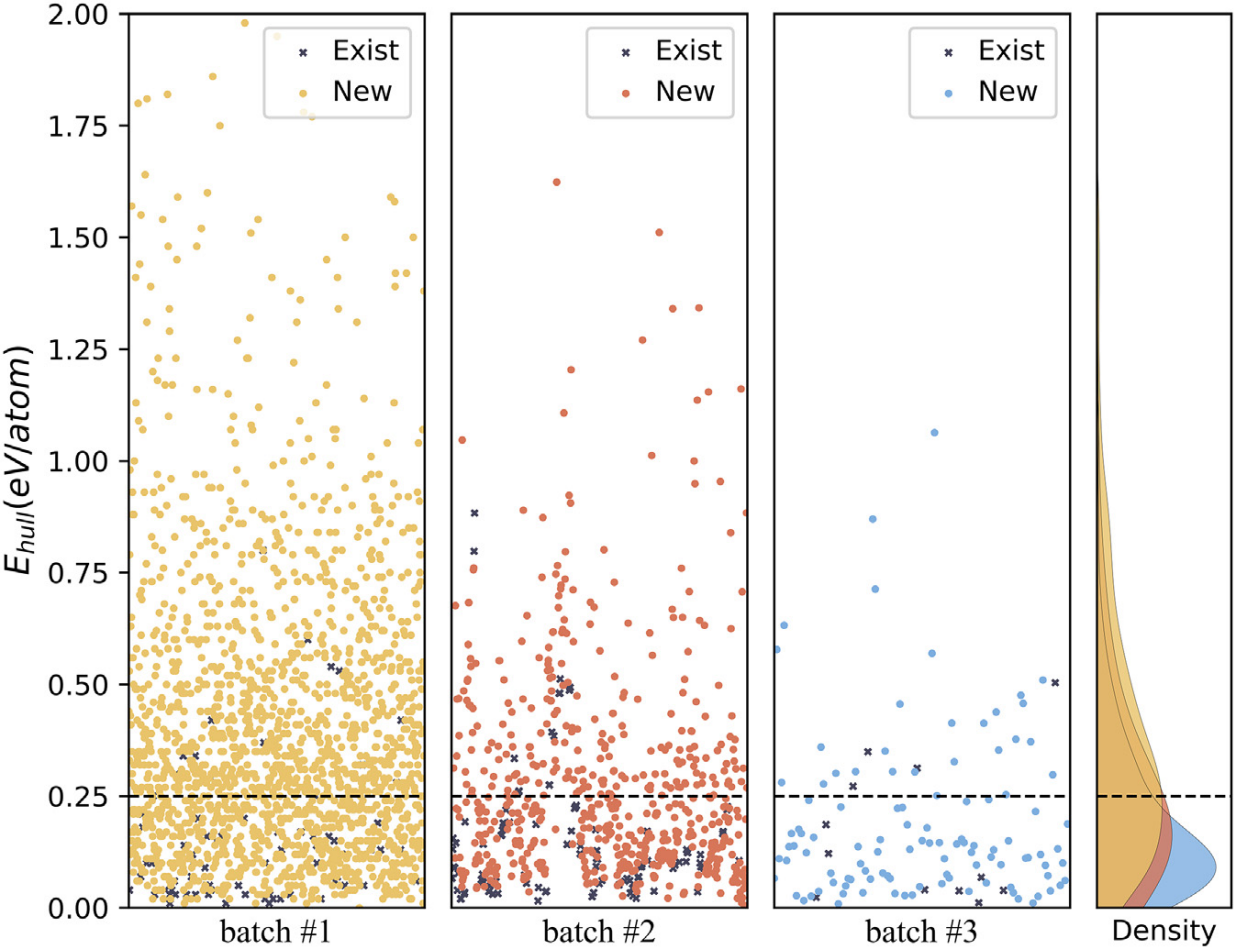
The distribution of E_hull_ per atom for the generated data (three batches) The label “Exist” means the generated structure is present in the database, while the label “New” indicates that it is outside of the database. Batch #1 contains structures with any components. Batch #2 contains structures that consist of rare earth elements, alkaline earth elements, transition metal elements, and oxygen. Batch #3 contains structures that consisted of common elements.

We employed the Vienna Ab initio Simulation Package (VASP)^29^^,^^30^ to calculate the total energy. The generalized gradient approximation (GGA)^31^ given by the Perdew–Burke–Ernzerhof (PBE) parametrization^32^ was used to describe exchange–correlation interactions. Furthermore, we utilized the *pymatgen* package to calculate the E_hull_ per atom^33^ and found that approximately 39.44%, 61.80% and 66.7% separately of the structures exhibited values less than 0.25 eV/atom (Figure 4 and Table 2). In comparison, according to Yong Zhao’s PGCGM,^4^ out of 1579 structures, 106 had values less than 0.25 eV/atom (5.3%). Sungwon Kim’s model^7^ and Juhwan Noh’s model, known as iMatGen^28^^,^ are two earlier models that have also made significant contributions to the field of GAN and VAE. Both of these authors assert that a structure with an E_hull_ less than 80 meV/atom can be considered relatively stable. In their respective paper, Sungwon Kim’s work obtained 113 results with an E_hull_ per atom less than 80 meV/atom from 6000 generated structures, while iMatGen achieved 40 such results from 10,981 structures, with a ratio of 1.8% and 0.36%, respectively. In contrast, we identified 160,122 and 41 structures that met these criteria from generated structures in these three batches (8.9%, 16.35%, 34.17%).

**Table 2:** Percent of structures with energy above the hull per atom lower than the given standard among these batches and some other models

E_hull_	Batch #1	Batch #2	Batch #2 (without rare earth elements)	Batch #3	PGCGM^4^	Sungwon Kim’s model^7^	iMatGen^28^
0.25 eV/atom	39.44%	61.80%	89.66%	66.67%	5.3%^4^	–	–
80 meV/atom	8.90%	16.35%	60.92%	34.17%	–	1.8%^7^	0.36%^28^

It is important to note that in batch #2, the definition and calculation method of E_hull_ may result in statistically anomalous values, either falsely high or low, particularly due to the limited representation of structures containing rare earth elements in the database. Consequently, we excluded structures containing rare earth elements from batch #2 and recalculated the statistics, as presented in Table 2. Further details can be found in the supplemental information. These findings suggest that the diffusion model may, to some extent, outperform GANs or VAEs in this field. It is worth highlighting that despite being a simplified version designed to explore the potential of the diffusion model and point cloud usage in the field of materials, our framework, akin to a pretrained model, has demonstrated comparable or even superior effectiveness in various aspects when compared to many other existing models.

**Figure 5:**
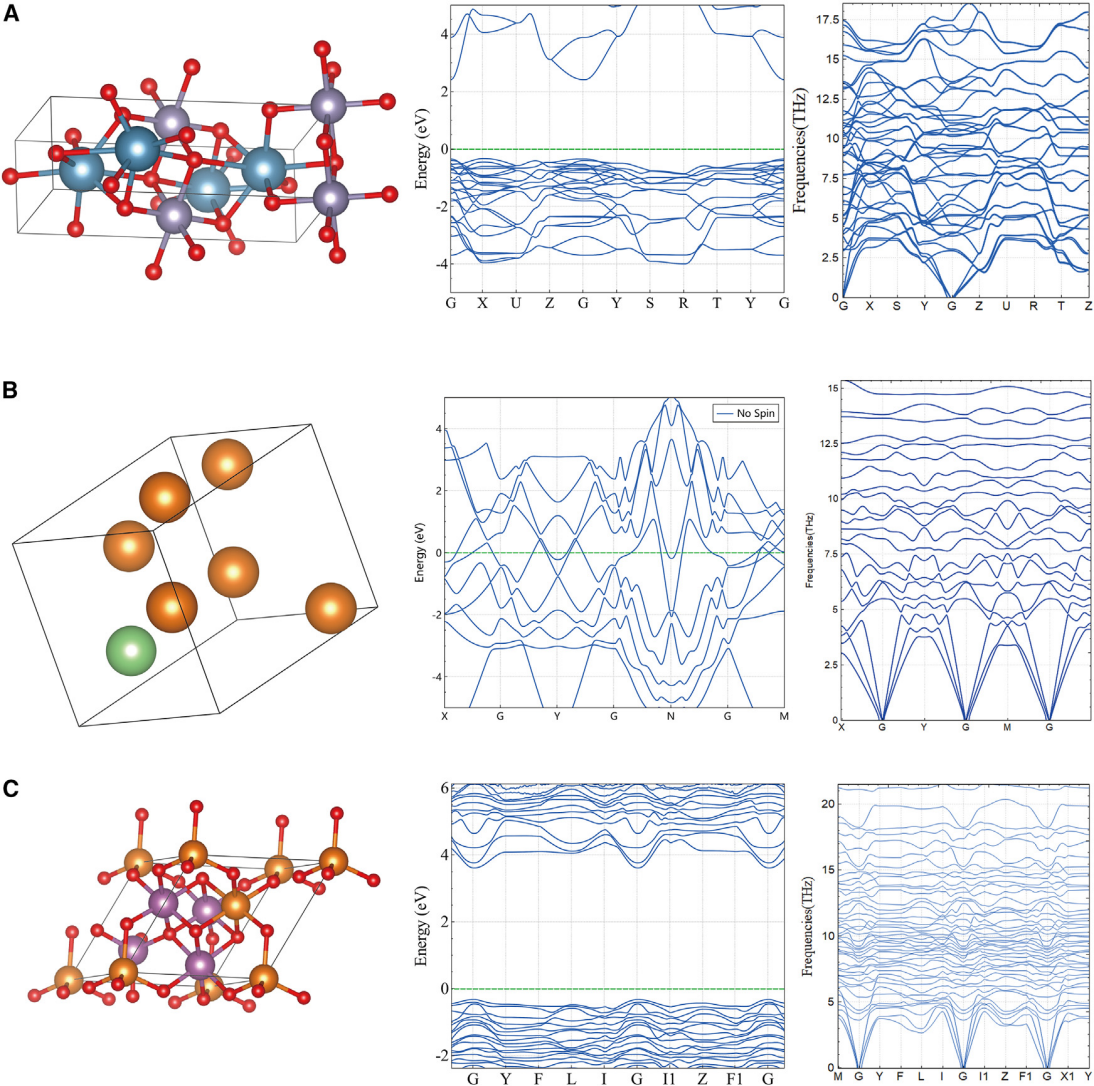
Graphical depiction of the structures and their DFT calculations Crystal representation and band and phonon band structures of Ca_2_SnO_4_ (A), LiMg_6_ (B), and MgSc_2_O_4_ (C).

Moreover, we chose three materials (Ca_2_SnO_4_, LiMg_6_, and MgSc_2_O_4_) from the generated structures for further investigation (as shown in Figure 5). We utilized DS-PAW^34^ for structural relaxation calculations and band structure assessments. Importantly, all three materials were successfully optimized via DFT calculations. Subsequently, we conducted phonon structure calculations for these selected materials, and all the materials demonstrated structural stability. Among these three compounds, MgSc₂O₄ and LiMg₆ are being not reported in MP database. These compounds are notably challenging to obtain through simple elemental substitution. Furthermore, among these three materials, MgSc_2_O_4_ and LiMg_6_ were reported, but they are difficult to obtain by simple elemental substitution. Furthermore, Cheng et al.,^35^ who used the PCCD to discover of Magnesium-Aluminum alloys, demonstrated that PCCD can generate structures effectively. This finding not only validates the use of PCCD in the discovery and design of materials but also opens avenues for future research in material science.

### Methods

**Figure 6:**
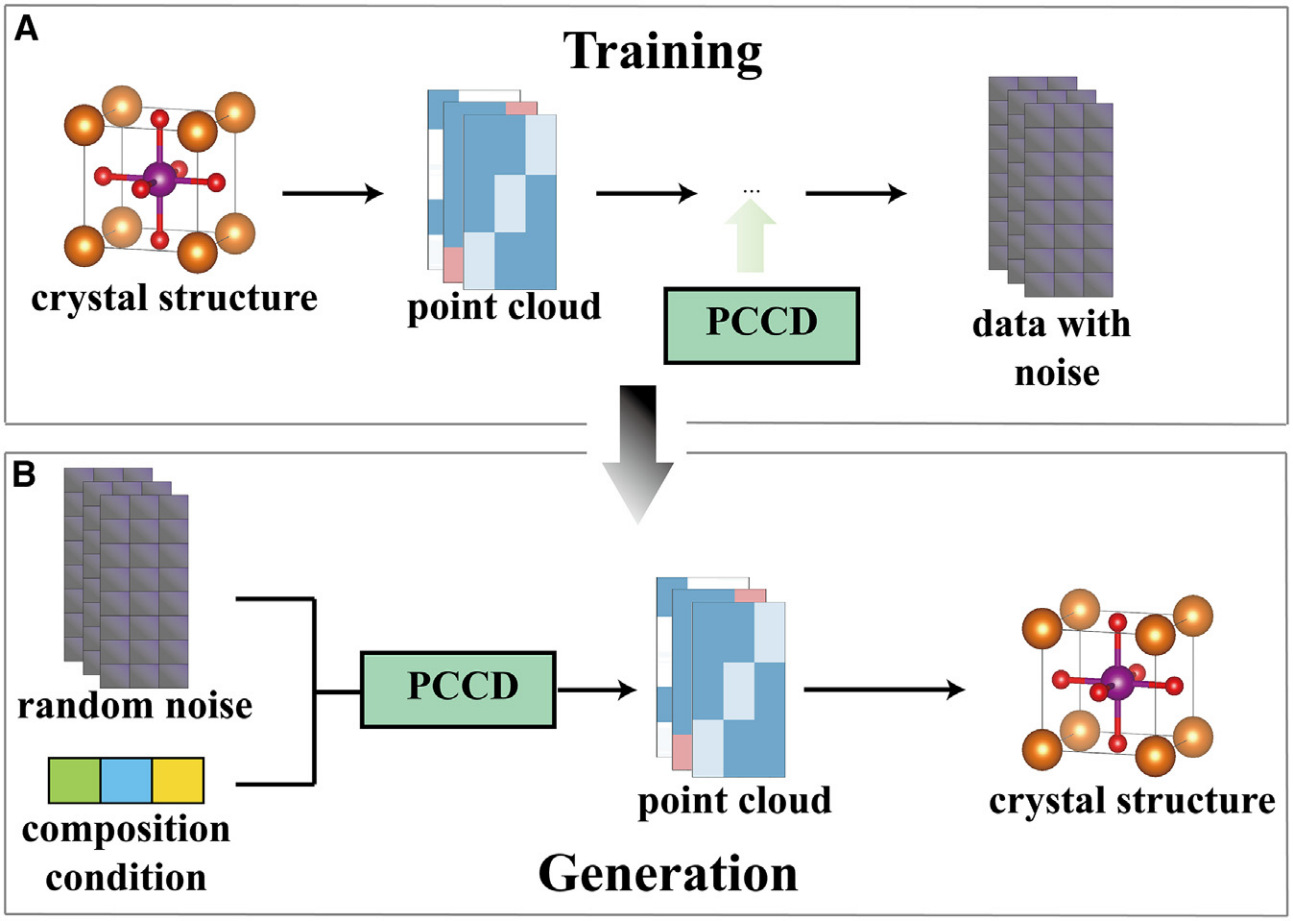
Sketch map of the PCCD (A) Training phase process with data manipulation section. First, crystals are transformed to a point cloud data type, followed by the addition of noise to the data, which enables the PCCD to perform observation and learning. (B) Generation phase with retrieval data operation. The method starts by feeding the PCCD random data and composition conditions and then passes to the data extraction and finishing with generating structures.

At the core of our approach is the utilization of a diffusion model as the foundational model, as illustrated in Figure 6. We leverage U-Net^36^ as the backbone of PCCD, a well-established architecture frequently employed for tasks such as classification and segmentation tasks.^37^^,^^38^

Two main methods are commonly used to represent a 3D object: voxels and point clouds. Voxel-based representation is thorough but resource intensive. In contrast, point clouds are more efficient than sparse matrices and reduce resource usage. Some prior works have claimed to use point clouds,^7^ but they essentially used individual points to lower computational costs. Our approach treats point clouds and lattice constants as three-channel entities akin to RGB in the computer vision (CV) field. We then employ clustering to determine the position, element composition and lattice.

Drawing from the notable achievements of diffusion models in the field of CV, we are motivated to extend their application to the generation of crystal structures. In this paradigm, we envision each crystal structure as akin to a patch in an image. To explore this innovative approach further, we integrate the point cloud representation technique with the power of diffusion models within the PCCD. This fusion of methods is designed to leverage the inherent advantages of both approaches. The diffusion model, renowned for its ability to capture intricate dependencies in data, holds promise for encoding the structural nuances of crystal formations. Moreover, the use of point cloud data representation, akin to a cloud of 3D points, serves to describe atomic positions and their attributes efficiently. By combining these two methodologies, we seek to harness their collective potential to revolutionize the generation and understanding of crystal structures.

#### Data preprocessing

Our material data were sourced from the Materials Project (MP).^2^ In this extensive database, our selection process targeted structures with ternary, binary, or monadic compositions that feature a maximum of 16 atom sites. This thorough filtering yielded a comprehensive dataset comprising 52,028 distinct materials. This dataset encompasses a wealth of information, including the POSCAR file, band gap, magnetism, crystal system, magnetic ordering, etc., for each of these structures. However, for model efficiency, we opted to narrow our focus to the band gap and magnetic ordering as the primary control variables. This decision, in conjunction with our use of the POSCAR files as training data, was made to streamline and lighten the model while ensuring the retention of essential variables for our specific research objectives.

**Figure 7:**
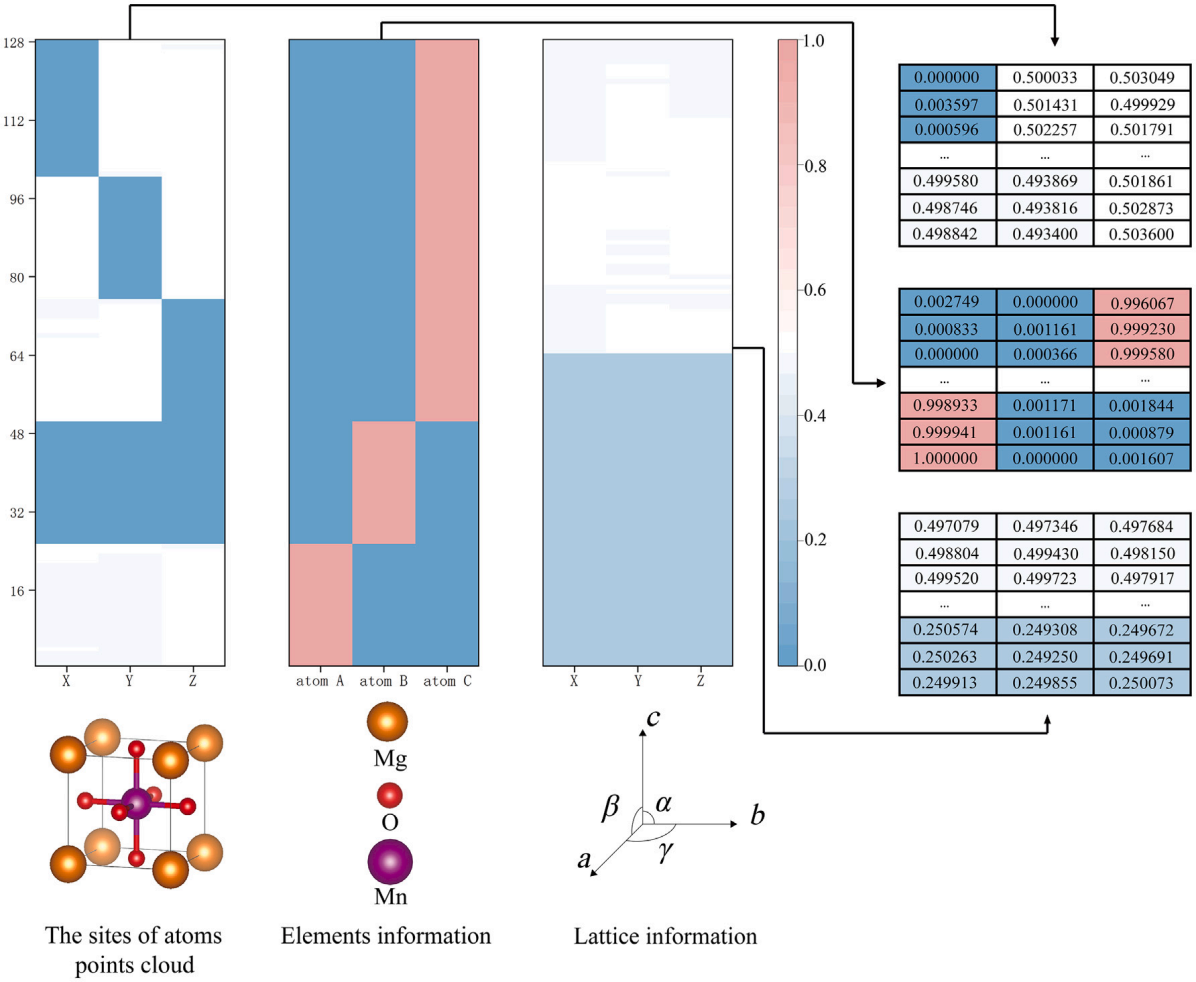
Data format for the framework (e.g., MgMnO_3_) The first channel represents the position, the second channel represents the element information, and the third channel determines the lattice constants.

As mentioned previously, we initially gathered various properties and POSCAR files of each crystal before training. The primary objective revolves around transforming the POSCAR data into a three-channel format, encompassing atom positions, element information, and lattice constants, as illustrated in Figure 7. Each of these channels comprises 128 items, effectively representing each structure as a 3×128×3 (C×W×H) matrix. The first channel is dedicated to atomic site information, where we distribute 128 points within the space. It is essential to clarify that the positions here are relative coordinates akin to those in the POSCAR file. The lattice vectors have not been determined at this stage. In essence, we use 128 items or several sets of data at this point in the process. To determine the absolute positions of these points, it is necessary to multiply them by the three lattice vectors obtained after processing the third channel. The data in the second channel correspond one-to-one with those in the first channel. Prior to generating or training samples, we input up to three elements. Each item in this channel contains three data values, which represent the likelihood of these three elements being associated with each atom. The data in the third channel do not correspond one-to-one with those in the first two channels. In fact, we want to obtain only six parameters α,β,γ,a,b,c from here, which can be converted to three vectors. To match the shape before, we expand them to 128 items by copying. In theory, after training, two distinct groups of data are generated. We can then obtain three vectors by employing clustering techniques, determining the means of each group, and performing calculations.

#### Generation model

Our generation model is based on the diffusion model, which is essentially a parameterized Markov chain. It is trained using variational inference to produce samples that closely match the data distribution after finite time.^20^

**Figure 8:**
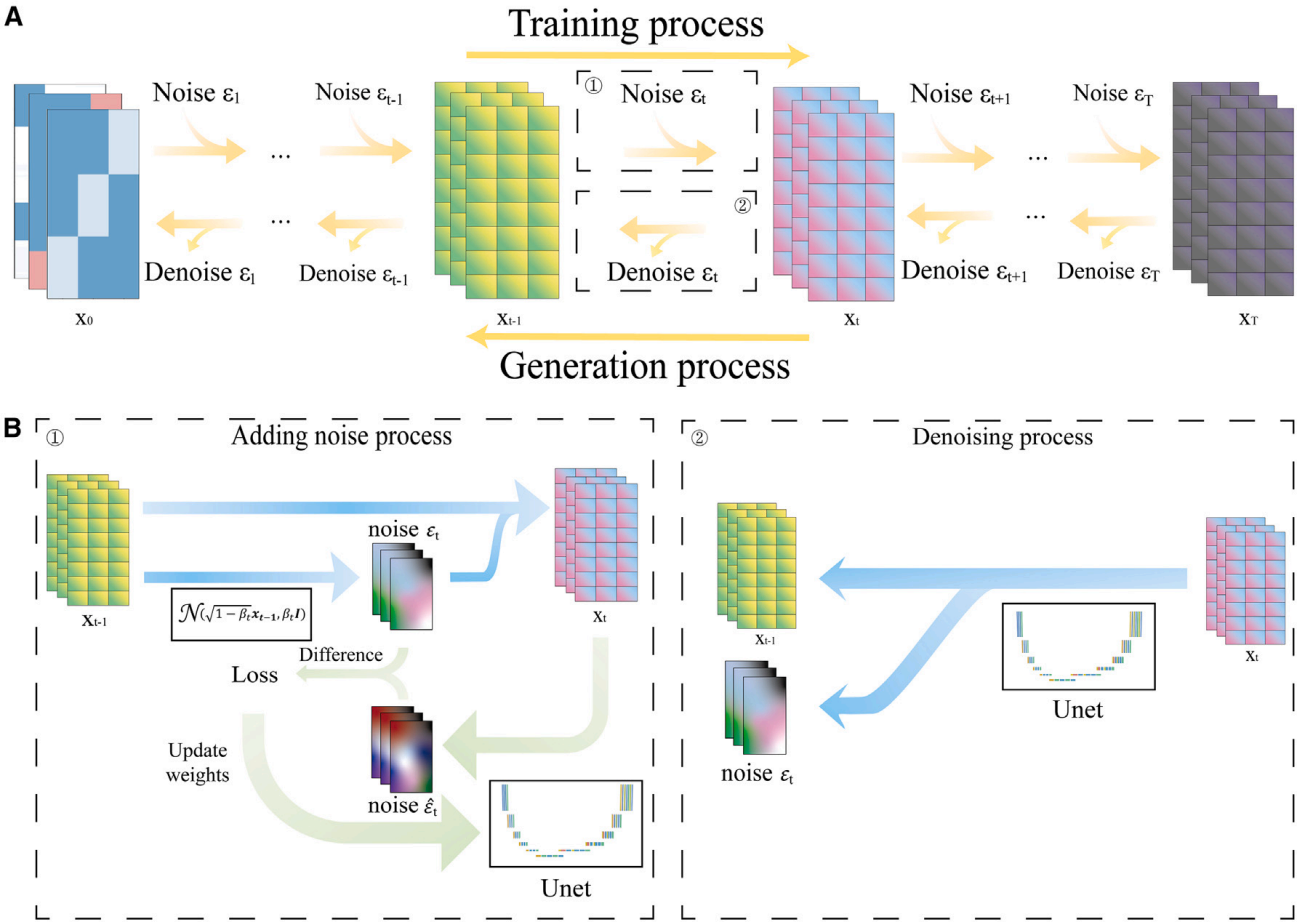
Schematic depiction of the PCCD architecture with generation and training processes (A) The data flow of the training process and generation process. (B①) A step in the model's training process corresponds to (A①). (B②) A step in the denoising process corresponds to (A②).

The diffusion model comprises two distinct processes, the training process and the generation process, often referred to as the sampling process, as illustrated in Figure 8A. These processes work in tandem to enable the generation of data samples that align with the underlying distribution of the training data. The training process can be briefly described as a procedure in which noise is progressively introduced to the data and the model endeavors to meet the characteristics of this noise addition. In contrast, the sampling process involves the gradual application of the trained model to denoise pure noise data. These data, in essence, are treated as source data with superimposed noise, and the model works to refine and clarify them.

The training process begins with $x_{0}$ and gradually adds noise $\varepsilon_{1},\varepsilon_{2},\cdots ,\varepsilon_{T-1},\varepsilon_{T}$ to $x_{0}$, resulting in $x_{1},x_{2},\cdots ,x_{T-1},x_{T}$. Assuming that $x_{0}∼q\left(x_{0}\right)$ and the noise $\varepsilon_{t}$ follow a normal distribution, then, for t≥1:

(Equation 1) $$q\left(x_{1:T}|x_{0}\right)=\prod_{t=1}^{T}q\left(x_{t}|x_{t-1}\right)\;q\left(x_{t}|x_{t-1}\right)=N\left(x_{t};\sqrt{1-\beta_{t}}x_{t-1},\beta_{t}I\right)$$

We follow the definition of J. Ho et al.^20^ Here, we define a constant variance schedule $\beta_{1},\cdots ,\beta_{T}$, where β increases as t increases. According to reparameterization, Equation 1 can also be expressed as:

(Equation 2) $$x_{t}=\sqrt{1-\beta_{t}}x_{t-1}+\sqrt{\beta_{t}\;}\;\epsilon$$

where ϵ∼N(0,1). We can obtain $x_{t}$ through the probability method from $x_{t-1}$. For simplicity, we define $\alpha_{t}=1-\beta_{t},\bar{\alpha_{t}}=\prod_{s=1}^{t}\;\alpha_{s}$ and $\bar{\beta_{t}}=\prod_{s=1}^{t}\beta_{s}$. By applying Equation 2 recursively, we can obtain that at any time t:

(Equation 3) $$x_{t}=\sqrt{\bar{\alpha_{t}}}x_{0}+\sqrt{1-\bar{\alpha_{t}}}\epsilon ,q\left(x_{t}|x_{0}\right)=N(x_{0};\sqrt{\bar{\alpha_{t}}}x_{0},\left(1-\bar{\alpha_{t}}\right)I$$

and the reverse process begins with $p\left(x_{T}\right)=N\left(x_{T},0,I\right)$; this process denoises gradually as $p_{\theta }\left(x_{0}\right)=\int p_{\theta }\left(x_{0:T}\right)dx_{1:T}$. In the reverse process $p_{\theta }$, we know the variance of every step but do not know the means $\mu_{\theta }$.

(Equation 4) $$p_{\theta }\left(x_{0:T}\right)=p\left(x_{T}\right)\prod_{t=1}^{T}p_{\theta }\left(x_{t-1}|x_{t}\;\right)\;p_{\theta }\left(x_{t}|x_{t-1}\right)=N\left(x_{t};\mu_{\theta }\left(x_{t},t\right),\beta_{t}I\right)$$

Therefore, we need to know what $\mu_{\theta }$ is. It can be derived that^20^^,^^39^:

(Equation 5) $$\mu_{\theta }\left(x_{t},t\right)=\frac{1}{\sqrt{\alpha_{t}}}\left(x_{t}-\frac{\beta_{t}}{\sqrt{1-{\bar{\alpha }}_{t}}}\;\epsilon_{\;\theta }\left(x_{t},t\right)\right)$$

After the parameterization (5), for any t∈[1,T]:

(Equation 6) $$x_{t-1}=\frac{1}{\sqrt{\alpha_{t}}}\left(x_{t}-\frac{\beta_{t}}{\sqrt{1-{\bar{\alpha }}_{t}}}\;\epsilon_{\theta }\left(x_{t},t\right)\right)+\sigma_{t}z$$

$\epsilon_{\theta }$ is the model that needs to be trained. This means that we can obtain $x_{t-1}$ from $x_{t}$ by $\epsilon_{\theta }$.

In a one-step noise addition process (Figure 8B①), the noise is composed of random numbers following a normal distribution. The mean and variance of this noise depend on time t and the preceding data $x_{\left(t-1\right)}$. Simultaneously, the PCCD actively learns the characteristics of this noise. During each iteration, the model receives data with noise, and its primary task is to predict the most recent noise addition. Consequently, we end up with two types of noise: one generated from a probabilistic approach and the other predicted by our deep learning model. By comparing these two noise sources, we can calculate a loss, which serves as feedback to the model, facilitating its adjustment and improvement. This iterative process continues until the model effectively learns to reproduce the noise characteristics, achieving accurate denoising.

In the noise-removal process, as shown in Figure 8B②, we only have data with noise, and our objective is to estimate and separate the noise from the data. In this way, we can separate the current noise and the previous data. At the macro level, this is a disorderly to orderly process (Figure S1).

In this context, we employ a U-Net model (Figure S2) to predict and separate noise from the data. U-Net, initially introduced in 2015,^36^ is a well-established model in the CV field that was notably acclaimed for its exceptional performance in image segmentation tasks. Our U-Net model is configured with five sets of upsampling and downsampling layers. To enhance its capacity to capture intrinsic data correlations, we incorporated intra-data correlation. This augmentation allows the model to effectively learn and predict noise, contributing to the denoising process.

### Conclusion

We introduced a framework employing the denoising diffusion probabilistic model (DDPM) and point cloud representation for crystal structure generation. This versatile framework enables the generation of crystal structures composed of fewer than three elements and featuring up to 16 atom sites by specifying the elemental composition. To assess the framework’s validity, we successfully reconstructed a batch of structures randomly sampled from the training dataset, confirming its reliability. Furthermore, we applied this framework to generate a batch of structures comprising rare earth elements, alkaline earth elements, transition metal elements and oxygen as an illustrative example. For the three batches of crystals generated by the PCCD, the percentages of structures with E_hull_/atom less than 0.25 eV/atom were 39.44%, 61.85% and 66.67%, respectively, and these with E_hull_/atom less than 80 meV/atom were 8.90%, 16.35% and 34.17%, respectively. Structures with some special components are more abundant. In addition, the stabilities of several structures have been confirmed through phonon structure analysis (e.g., Ca_2_SnO_4_, LiMg_6_, and MgSc_2_O_4_). Consequently, we demonstrated the efficacy of utilizing DDPM and point cloud representations in crystal structure generation, which was validated by DFT high-throughput calculations. This framework serves as a foundational step, offering potential for further enhancement and the development of larger models for inverse crystal design. Furthermore, this approach serves to expand the database of crystals.

### Limitations of the study

The constraints of PCCD are still few and cannot achieve true controllable generation yet. Introducing spatial group constraints to explore the chemical space of materials may be a valuable approach, not only to enhance the validity, novelty, and stability of the generated materials but also to improve the efficiency and effectiveness of the generative process in future research. Furthermore, we need to advance the model in the future to support inverse design capabilities for a broader range of chemical elements (beyond three), desired material properties, and experimental observations. This development could facilitate the systematic exploration and discovery of materials with targeted functionalities, thereby addressing key challenges in materials science.

## Resource availability

### Lead contact

Further information and requests for resources should be directed to and will be fulfilled by the lead contact, Shibing Chu (c@ujs.edu.cn).

### Materials availability

This study did not generate new unique reagents.

### Data and code availability

• This paper analyzes existing, publicly available data. The accession information for these datasets are listed in the key resources table.
• All original code has been deposited at the GitHub repository (https://github.com/lzhelin/CrystalDiffusion) and is publicly available.
• All additional information required to reanalyze the data reported in this paper is available from the lead contact upon request.

## Acknowledgments

This work gratefully acknowledges the 10.13039/501100001809National Natural Science Foundation of China (No. 11904137, 12074150 and 12174157) and the financial support from 10.13039/501100002703Jiangsu University (No. 4111190003). We gratefully acknowledge HZWTECH for providing computation facilities.

## Author contributions

Z.L.: conceptualization, method, software, investigation, formal analysis, model validation, writing original draft; R.M.: editing, model validation and investigation; R.J., G.H., and J.S.: DFT calculation guide; S.C. and Y.C.: conceptualization, writing review, funding acquisition, resources and supervision.

## Declaration of interests

The authors declare no competing interests.

## STAR★Methods

### Key resources table

**Table:** 

REAGENT or RESOURCE	SOURCE	IDENTIFIER
**Deposited data**		

The Materials Project	Jain et al.^2^	https://doi.org/10.1063/1.4812323 https://next-gen.materialsproject.org/

**Software and algorithms**		

CrystalDiffusion	This paper; original code for reported results.	https://github.com/lzhelin/CrystalDiffusion https://zenodo.org/records/10570395
CSPBenchMetrics	Wei et al.^27^	https://doi.org/10.1016/j.commatsci.2024.112802. https://github.com/usccolumbia/CSPBenchMetrics
VASP	VASP Software GmbH	https://www.vasp.at/

### Method details

#### Data collection

For this study, we curated the dataset from Materials Project, focusing on structures with ternary, binary, or monadic compositions and limiting the number of atomic sites to a maximum of 16. This filtering process resulted in a dataset of 52,028 materials. Each entry in this dataset includes detailed information such as the POSCAR file, band gap, magnetic properties, crystal system, and magnetic ordering, among other key attributes.

#### Model training details

In PCCD, we employ the U-Net for noise prediction (Figure S2). It primarily encompasses four up-sampling progresses and for down-sampling progresses, with each progresses comprising multiple convolutional layers and self-attention layers.

As depicted in Equation 3, for each step t during the training progress, we can calculate $x_{t}$, while the noise $\epsilon_{t}∼N\left(0,1\right)$ is given. The objective of the U-Net is to estimate the noise term $\epsilon_{t}$ given $x_{t}$. For the loss of U-Net (Figure S3), we utilize Mean Absolute Error (MAE, Equation 7) to quantify the discrepancy between output of U-Net and $\epsilon_{t}$. For more Hyperparameters details in training can be seen in Table S1, supplemental information.

While training, we use Mean Absolute Error (MAE) to calculate loss (Figure S3). The loss function is as follow.

(Equation 7) $$Loss=E_{t,x_{0},\epsilon }\left(\left\|\epsilon -\epsilon_{\theta }\left(\sqrt{{\bar{\alpha }}_{t}}x_{0}+\sqrt{{1-\bar{\alpha }}_{t}}\epsilon ,t\right)\right\|\right)$$

Here, t is timestep, ${\;x}_{0}$ is the original data (training data) without noise, ϵ is random matrices with the same shape of ${\;x}_{0}$ following normal distribution.

#### Data expression example

Taking MgMnO_3_ as an example (Figure 7), the data is generated by our diffusion model. For the first channel, obviously, 128 data points can be classified into five categories: (0, 0.5, 0.5), (0.5, 0, 0.5), (0.5, 0.5, 0), (0, 0, 0) and (0.5, 0.5, 0.5). The clustering method we use is Density-Based Spatial Clustering of Applications with Noise (DBSCAN) due to the raison of undefined number of groups in our data. However, here we may know: one atom is at the position of (0, 0, 0), one atom is at the center of the crystal body, and three atoms are at the centers of the crystal faces. However, we have not been given more information on elements and lattice. It can be a carbon system or a Ca-Ti-O system, and can also be a triclinic system or a hexagonal system. At the second channel, it gives the element information for the 128 points one by one. In Figure 7, it can be seen that there are three groups of data, further categorize the five categories in the first channel into three by one-to-one correspondence”, we change like follow “the first data channel is divided to five classes of data while the second is divided to three classes. However, these two channels (first and second) are aligned according to three classes of data (second channel) due to index correspondence (see how classes are aligned in Figure 7). and they further categorize the five categories in the first channel into three by one-to-one correspondence. So far, the elements and the relative coordinate in unit cell of every atom can be confirmed. Before training, the elements information have been given by inputting a list (Mg, Mn, O), which assign in the second channel: (1,0,0), (0,1,0), and (0,0,1) mean Mg, Mn, O, separately. But the shape of lattice or crystal system is still unknown, and it can also be a cubic system or a trigonal system. Ignoring the first two channels, while processing the third channel, we deal with them directly. It can be aggregated into two categories theoretically which are the lenghth a,b,c and angle α,β,γ of lattice. During training and data preprocess, all samples have the same template for the third channel. Hence, we neglect the use of additional clustering algorithm, only perform calculations average for every column of the front half (α,β and γ) and back half (a, b and c). Here, the data we get approximately are (0.50, 0.50, 0.50), (0.25, 0.25, 0.25) (All numbers below 1 is because before training, lattice data have been normalized by dividing 15Å and the angle used radian system and divided by 2π ). Then, the three vectors of the lattice can be calculated as follows.

(Equation 8) $$\vec{a}=a\left(1,0,0\right)$$

(Equation 9) $$\vec{b}=b\left(\left(\cos\;\gamma ,\sin\;\gamma ,0\right)\right)$$

(Equation 10) $$\vec{c}=c(\cos\;\beta ,\cos\;\alpha -\frac{\cos\;\beta \;\cos\;\gamma }{\sin\;\gamma },\frac{\sqrt{1+2\;\cos\;\alpha \;\cos\;\beta \;\cos\;\gamma -\cos^{2}\;\alpha -\cos^{2}\;\beta -\cos^{2}\;\gamma }}{\sin\;\gamma }$$

The result is (3.75, 0, 0), (0, 3.75, 0), (0, 0, 3.75), and further determine that it’s a cubic system with side length of 3.75Å. Integrate the above all, we summarize:

1. It’s a cubic system with side length of 3.75Å.
2. The formula of this structure is MgMnO_3_.
3. For every unit cell, there will be an oxygen atom on each face, a magnesium atom on each corner, and a manganese atom at the center.

#### Generation processes example

Also taking MgMnO_3_ as an example(Figure S1), different color of points means different atoms (the second channel of data) and ignoring the third channel for better visualization. The whole inference process need 1000 steps. It’s the last step for training and also the first step for generation while t=999, while generating, the data given here is random numbers obey a normal distribution. It can be seen that the points in this step are disorganized. with t getting closer and closer to 0. Points with the same color gradualy come together. From t=200, there are already rudiments of clustering. While t=0, it can be clear that there are five groups, and it means that this structure has these five atoms, including positions and elements.

#### DFT configuration

The structures were optimized by Density Functional Theory (DFT) that were carried out with Vienna ab initio simulation package (VASP). The Perdew–Burke–Ernzerhof (PBE) of the generalized gradient approximation (GGA) was used for exchange–correlation functional. The kinetic energy cutoff was set to be 520 eV for the electronic wavefunction having a plane wave basis set which was obtained using the projector augmented-wave method. The Monkhorst–pack k-mesh grids was selected by vaspkit.

### Quantification and statistical analysis

There are no quantification or statistical analyses to include in this paper.
