# Supplementary material for: Generative design of crystal structures by point cloud representations and diffusion model
Source: iScience. 2024 Dec 20;28(1):111659. doi: 10.1016/j.isci.2024.111659 (PMC11763582; doi:10.1016/j.isci.2024.111659)
Supplement: Document S1. Figures S1–S3 and Table S1 [file mmc1.pdf]

iScience, Volume *28*

Supplemental information

Generative design of crystal structures by point cloud representations and diffusion model

Zhelin Li, Rami Mrad, Runxian Jiao, Guan Huang, Jun Shan, Shibing Chu, and Yuanping Chen

Supplemental Information

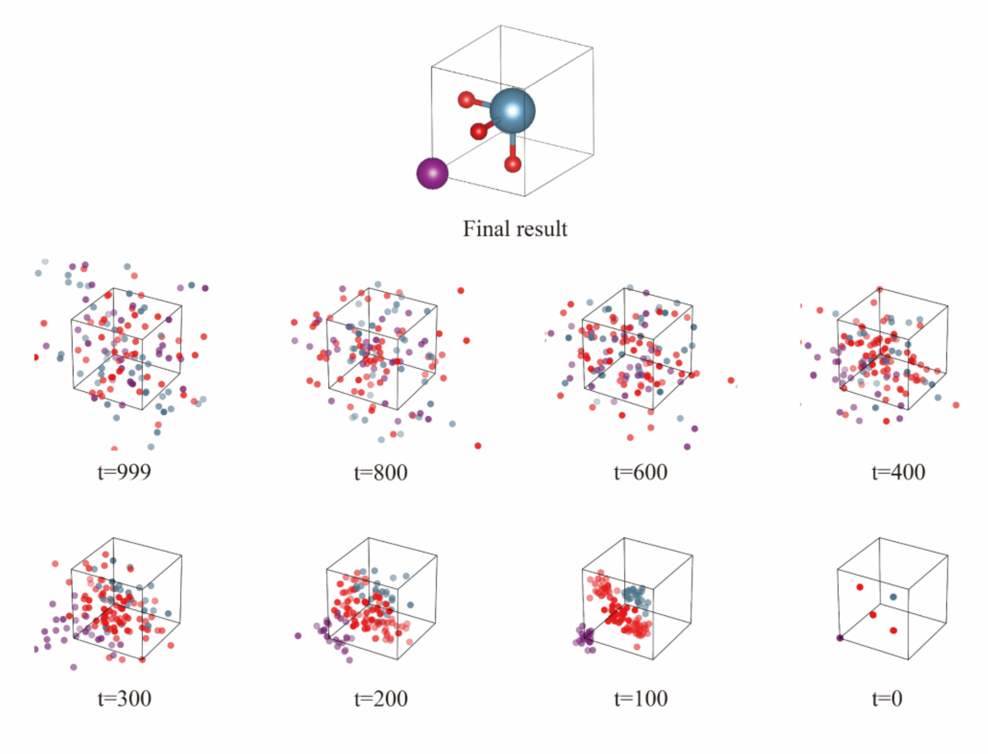

Fig S1. Generation processes example.

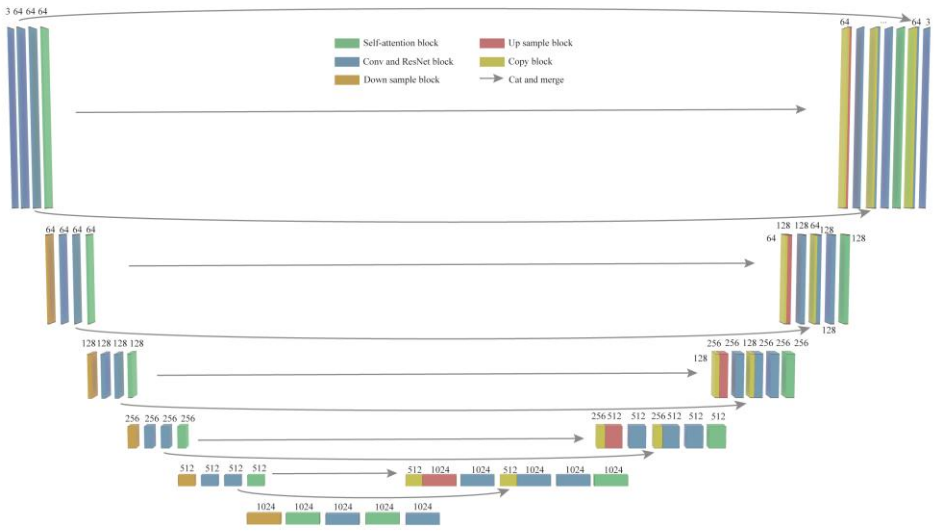

Fig S2. U-Net model used in diffusion.

Hyperparameter	Value
Optimizer	Adam ($\beta_1 = 0.9,\ \beta_2 = 0.99$)
Learning rate	0.0001
Batch size	128
Training steps	500
Prediction target	ϵ
Sampling timesteps	1000
Diffusion noise schedule	cosine
Training dataset samples	51,212
Testing dataset samples	868

Table S1. Hyperparameters for training.

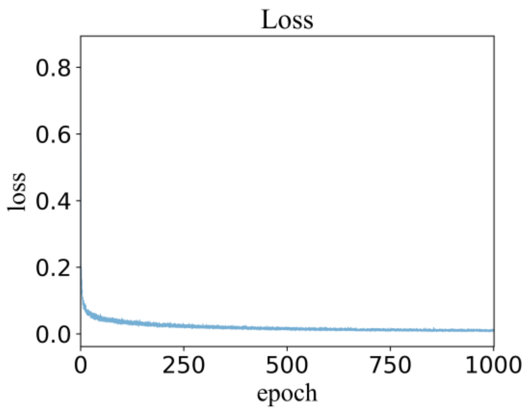

Fig. S3 Loss plot.
